# 3D printed eyeball implants in dogs with advanced glaucoma: a case series study

**DOI:** 10.3389/fvets.2025.1712326

**Published:** 2025-12-10

**Authors:** Joelson Cavalcanti Silva, Lucas Rannier Ribeiro Antonino Carvalho

**Affiliations:** 1 Postgraduate Program in Veterinary Ophthalmology, ANCLIVEPA-SP, São Paulo, Brazil; 2 Department of Physiology and Pharmacology, Karolinska Institutet, Stockholm, Sweden

**Keywords:** 3D technology, glaucoma, eyeball, veterinary ophthalmology, polylactic acid

## Abstract

Glaucoma is a chronic and progressive ophthalmic disease in dogs, characterized by increased intraocular pressure (IOP), leading to severe pain, irreversible blindness, and impaired quality of life. In advanced stages, enucleation is recommended as a definitive treatment for pain relief. This study describes surgical procedures for ocular enucleation associated with 3D printed eyeball implants in 11 dogs with chronic refractory glaucoma. All procedures were performed by the Ophthalmology Department of the SouVet Veterinary Hospital in João Pessoa-Brazil. The animals were aged between 4 and 16 years, presented buphthalmos, persistent intraocular hypertension, and no longer responded to conventional clinical treatments. Enucleation surgery was performed through a transconjunctival incision, with removal of the eyeball and implantation of the 3D printed prosthesis produced with biocompatible thermoplastic polylactic acid (PLA). In addition to the benefits of enucleation for the management of advanced glaucoma, replacing the eyeball with a 3D prosthesis of the same volume and weight offered greater comfort to the patient and improved post-surgical adaptation. The eye cavity was better protected, reducing the risk of accumulation of secretions and infection, in addition to better preventing eyelid deformities. It was also possible to observe an exponential improvement in the emotional wellbeing of the animal guardians. In the cases described, no inflammatory complications or signs of rejection of the 3D printed eyeball prosthesis were observed over 8 months of post-operative evaluation. The use of biocompatible materials and 3D printing for personalizing the prostheses provided a satisfactory aesthetic result and a recovery without significant complications. Further studies are needed to evaluate the long-term safety of this option for the management of advanced glaucoma.

## Introduction

Glaucoma is a chronic and progressive ophthalmic disease characterized by increased intraocular pressure (IOP), which leads to irreversible damage to the optic nerve and permanent vision loss. In dogs, glaucoma can be classified as primary or secondary (1). Primary glaucoma is related to anatomical anomalies in the aqueous humor drainage system, such as trabecular atrophy or failures in the production of the iridocorneal angle, while secondary glaucoma is caused by inflammatory processes, trauma, lens dislocation or intraocular neoplasms (1). Uncontrolled increased IOP results in degeneration of the optic nerve and, consequently, loss of vision, with progressive worsening of the condition (2).

Physiologically, aqueous humor is produced by the ciliary processes and normally drained through the trabecular meshwork and the sclerocorneal network. In glaucoma, this drainage flow is compromised, leading to the accumulation of aqueous humor inside the eye and, consequently, to an increase in IOP. If this pressure is not reduced, the optic nerve is compressed, which can result in irreversible damage to the nerve fibers, leading to blindness (1).

Regarding the causes, primary glaucoma is associated with a genetic predisposition and is commonly observed in breeds such as Cocker Spaniel, Basset Hound, and Poodle, which present anatomical alterations in the iridocorneal angle, impairing aqueous humor drainage (3). Genetic factors play a significant role in the predisposition of certain breeds, such as Basset Hound, to develop the disease (4). Secondary glaucoma, which is more common, is triggered by several ocular conditions, including uveitis, lens dislocation, neoplasms or intraocular hemorrhages (5–7).

The diagnosis of canine glaucoma is based on a combination of clinical findings and complementary exams. The most common clinical signs include eye pain, excessive tearing, photophobia, corneal opacity, and an enlargement of the eyeball, a condition known as buphthalmos (1). Assessment of intraocular pressure is essential for diagnosis and is performed by tonometry, using devices such as the applanation tonometer or the rebound tonometer (8).

In addition to tonometry, it is essential to perform examinations of the fundus of the eye, such as ophthalmoscopy, to observe the health of the optic nerve. Fundoscopy can also reveal atrophy of the optic disc and cupping of the optic nerve, which are indicative of damage due to increased IOP (1). Clinical treatment of canine glaucoma aims to reduce intraocular pressure and relieve pain. Medications frequently used include beta-blockers (such as timolol), carbonic anhydrase inhibitors (such as dorzolamide), and alpha-adrenergic agonists (5). These agents act to decrease the production of aqueous humor or increase its drainage. In addition, anti-inflammatories and analgesics are often used to control ocular pain and associated inflammation (2).

Even with accurate diagnosis and clinical treatment, many cases of glaucoma become refractory, especially in more advanced stages of the disease, when the optic nerve is already severely compromised (9, 10). In these cases, surgical intervention is often necessary; goniovalvuloplasty and transscleral cyclophotocoagulation (TSCPC) are surgical techniques used to control intraocular pressure. Goniovalvuloplasty aims to restore the drainage flow of aqueous humor through incisions in the iridocorneal angle, while TSCPC uses a laser to destroy part of the ciliary processes, decreasing the production of aqueous humor (2). However, in cases of irreversible and refractory glaucoma, enucleation (removal of the eyeball) is a therapeutic option to relieve chronic pain and avoid additional complications.

Enucleation can also be associated with the implantation of prostheses for volumetric replacement of the eyeball, with the aim of reducing enophthalmos, eyelid retraction and the risk of accumulation of secretions. In addition, the prostheses improve the aesthetic appearance of the animal and provide emotional benefits for the owners.

Three-dimensional (3D) printing technology to produce custom models has great potential for applications in ophthalmology (11). In addition to the production of prostheses for eyeball replacement (12), 3D models can be used to evaluate the correlation between different corneal thicknesses, for simulation of intraocular pressure as an alternative to conventional porcine eye models (13), for teaching direct ophthalmoscopy, surgical planning, and other applications (14, 31, 36).

Various methods and materials can be applied in 3D printing, spanning a wide range of fields such as metal fabrication, resin-based manufacturing, civil engineering, the food industry, and tissue or cellular engineering. Among these techniques, fused deposition modeling (FDM) is one of the most commonly employed worldwide due to its accessibility, cost-effectiveness, and ability to produce complex geometries. In this method, a thermoplastic filament is heated and extruded layer by layer to build a three-dimensional structure.

The most frequently used materials in FDM include polylactic acid (PLA), acrylonitrile butadiene styrene (ABS), polyethylene terephthalate glycol (PETG), and carbon fiber–reinforced composites, each providing distinct mechanical strength, flexibility, and thermal stability. Additionally, flexible filaments such as thermoplastic polyurethane (TPU) and thermoplastic elastomers (TPE) have gained increasing attention for applications that require elasticity and impact absorption, including soft-tissue modeling and cushioning structures (11, 15).

Polylactic acid (PLA), in particular, stands out among biodegradable polymers due to its excellent biocompatibility, ease of printing, and mechanical rigidity. Compared to other resorbable materials such as polyglycolic acid (PGA) and polycaprolactone (PCL), PLA is also the most easily accessible. These combined features, along with its smooth surface finish and cost-effectiveness, make PLA a reliable choice for 3D-printed ocular implants in veterinary practice (14, 16, 37).

Facing the need for new approaches and protocols to improve the quality of life of patients with severe ophthalmological diseases and the lack of studies of this nature applied to veterinary medicine, this study aims to describe the case series of 11 dogs with chronic refractory glaucoma, which underwent enucleation with implantation of a biocompatible 3D eyeball prosthesis.

## Methods

All procedures involving animals in this study were performed by the Ophthalmology Department of the SouVet Veterinary Hospital in João Pessoa, Brazil. The modeling and manufacturing of the 3D prostheses was performed in collaboration with the Brazilian start-up 3D Medicine (@3dmedicine.br).

The study began in April 2024 and until the present writing of this report, the animals are still being monitored periodically. The indication for the surgical procedure and implantation of the 3D prosthesis was based on specific criteria, including chronicity, failure to respond to previous clinical treatments, presence of severe pain, buphthalmos, and irreversible blindness. This series of reports included 11 dogs of different breeds, ages (ranging from 4 to 16 years) and sexes, totaling 12 affected eyes. The clinical parameters of the first evaluation and epidemiological information are described in Table 1.

**Table 1:** Demographic description and clinical parameters of dogs with advanced glaucoma.

**Case number**	**Age (years)**	**Gender**	**Breed**	**Weight (kg)**	**HR (bpm)**	**RR (mpm)**	**Temp (°C)**	**SAP (mmHg)**	**Behavior aspect**
**1**	4.5	M	French Bulldog	8.6	160	50	38.4	150	Apathetic
**2**	13.9	M	Pinscher	2	180	60	38.9	130	Apathetic
**3**	10.2	F	French Bulldog	16.75	170	44	38	120	Apathetic
**4**	11.1	M	German Shepherd	40.2	120	37	39.1	130	Apathetic
**5**	11.5	F	Shih- Tzu	9.35	170	25	38.2	120	Apathetic
**6**	9.8	F	Pinscher	2.85	160	56	38.1	160	Apathetic
**7**	5.3	F	Poodle	7.5	140	48	39.6	150	Apathetic
**8**	16.2	M	Shih-Tz	6.6	150	36	38.6	130	Apathetic
**9**	8.5	F	Shih-tzu	15.7	160	24	39.1	120	Apathetic
**10**	5.4	F	Mixed breed	16.2	190	70	40.1	180	Apathetic
**11**	10.9	M	Mixed breed	9.2	140	29	38.7	140	Apathetic
**Mean** **±SD**	**9.75** **±3.4**	–	–	**12.26** **±10.5**	**158.18** **±19.9**	**43.54** **±14.9**	**38.8** **±0.6**	**139.09** **±19.2**	–

HR, Heart rate; RR, Respiratory rate; SAP, Systolic blood pressure.

After clinical evaluation and referral to the ophthalmology department, all dogs underwent a complete ophthalmic examination (Table 2), which included echobiometry, assessment of pupillary reflexes, vision analysis, Schirmer tear test and tonometry with a FA-800 VET rebound tonometer (Fuan Enterprise, Shanghai, China). All tests were performed according to the ophthalmic evaluation protocols in dogs with glaucoma (1). The ocular globe symmetry was classified as: “Buphthalmic” when an abnormal enlargement of the eyeball was observed, and “No changes” when the eye showed no evident clinical alterations (Table 2). The transcorneal echobiometry technique used to obtain the ultrasound image in B mode (brightness), with the patient in sternal recumbency and using a high-frequency linear transducer (15 MHz), conductive gel and prior anesthetic eye drops (17). The results of the echobiometry were described in Table 3. During the examination, it was also observed that the eyes with increased intraocular pressure (IOP) presented corneal edema, congested sclera, and mydriasis.

**Table 2:** Ophthalmological parameters of dogs with advanced glaucoma.

**Case number**	**Progression**	**Eyeball symmetry**		**Pupillary reflex**		**STT (mm/min)**		**IOP (mmHg)**	
		**RE**	**LE**	**RE**	**LE**	**RE**	**LE**	**RE**	**LE**
**1**	Chronic	*Buphthalmic*	No changes	–	+	0	15	54	23
**2**	Chronic	No changes	*Buphthalmic*	–	–	14	9	20	33
**3**	Chronic	*Buphthalmic*	No changes	–	+	8	15	41	20
**4**	Chronic	No changes	*Buphthalmic*	+	–	16	6	20	36
**5**	Chronic	No changes	*Buphthalmic*	+	–	15	0	19	40
**6**	Chronic	No changes	*Buphthalmic*	+	–	17	10	22	34
**7**	Chronic	*Buphthalmic*	No changes	–	+	5	12	–	16
**8**	Chronic	*Buphthalmic*	No changes	–	+	0	16	47	22
**9**	Chronic	No changes	*Buphthalmic*	+	–	13	3	19	35
**10**	Chronic	*Buphthalmic*	*Buphthalmic*	–	–	11	9	53	47
**11**	Chronic	*Buphthalmic*	No changes	–	+	3	14	–	20

RE, Right eye; LE, Left eye; STT, Schirmer tear test; IOP, Intraocular pressure.

**Table 3:** Results of echobiometry by B-mode ultrasound (Brightness) in dogs with advanced glaucoma.

**Case number**	**Eyeball diameter (cm)**		**Anterior chamber depth (cm)**		**Lens axial length (cm)**		**Vitreous chamber (cm)**	
	**RE**	**LE**	**RE**	**LE**	**RE**	**LE**	**RE**	**LE**
**1**	3.01	2.26	0.76	0.37	0.75	0.72	1.30	0.96
**2**	2.10	2.53	0.33	0.61	0.70	0.71	0.80	1.0
**3**	2.65	2.23	0.71	0.33	0.74	0.70	1.0	0.98
**4**	2.17	2.52	0.27	0.36	0.80	0.86	0.90	1.11
**5**	2.09	2.49	0.31	0.54	0.71	0.72	0.73	1.02
**6**	1.89	2.04	0.19	0.25	0.72	0.72	0.80	0.81
**7**	2.59	2.09	0.55	0.24	0.74	0.65	1.1	0.91
**8**	2.37	2.08	0.43	0.21	0.70	0.67	1.0	0.94
**9**	2.18	2.51	0.14	0.55	0.73	0.86	0.87	1.08
**10**	2.45	2.49	0.47	0.51	0.81	0.84	1.03	1.1
**11**	2.50	2.12	0.44	0.15	0.74	0.71	1.2	0.93

RE, Right eye; LE, Left eye.

The patients in this report were referred for specialized surgical treatment after unsuccessful attempts with conventional clinical therapies, which included the use of anti-inflammatory, antibacterial and hypotensive eye drops such as Brinzolamide + Timolol Maleate, which are widely used to control ocular hypertension in dogs, as described in the literature (1, 18). The tutors of the animals were duly informed about the surgical procedure with implantation of the 3D prosthesis and agreed to the procedure through the free and informed consent form. Prior to the surgical procedure, all animals underwent a general clinical evaluation, cardiological, hematological and echocardiographic examinations, to ensure suitability for the procedure.

**Figure 1:**
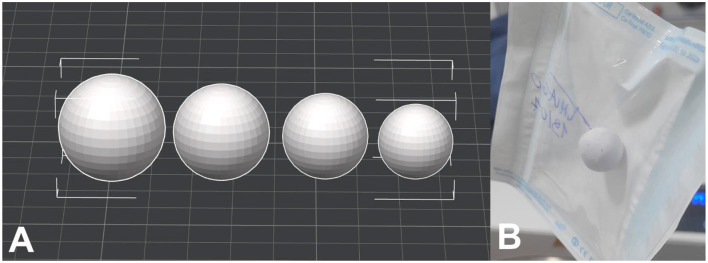
**(A)** Virtual model of 3D prostheses in 4 different sizes imported into the BambuStudio slicing software. **(B)** 3D printed ocular PLA prosthesis after autoclaving prior to surgical implantation.

During surgical planning, the morphometric data of the eyeball measured by echobiometry (Table 3) were used for 3D modeling of the custom-made prostheses using the Blender^®^ software version 3.6 (19). The virtual models of the prostheses were transferred to the BambuStudio^®^ slicing software version 1.10.2 (BambuLab, Shenzhen, China) to prepare for 3D printing by FDM using the X1 Carbon Combo printer (BambuLab, Shenzhen, China) (Figure 1A).

The ocular prostheses were printed using polylactic acid (PLA) filaments, a biodegradable and low-cost material widely used in 3D printing. PLA is a thermoplastic derived from renewable sources, such as cornstarch, and was used in this study because it offers good biocompatibility and wear resistance, when compared to other filaments used by FDM printers, such as ABS (Acrylonitrile Butadiene Styrene) and PETG (Polyethylene Terephthalate Glycol).

For 3D printing the protocol adapted from Carvalho (15) was used, with the following main parameters: layer height of 0.12 mm, 100% infill, grid supports, printing speed of 200 mm/s, printing temperature of 210 °C, with a heated table at 55 °C. After 3D printing and removal of the supports, the prosthesis was sanded for a better surface finish and immersed in 70% alcohol for 2 hours. After drying, the prostheses were sent for sterilization by autoclaving and stored in a cool place protected from light until surgical implantation in the animals.

Different autoclaving protocols were tested to ensure satisfactory sterilization of the material without deformation (data not shown). Even though PLA has a glass transition temperature (Tg) around 60–65 °C, the autoclaving protocols using a temperature of 170 °C for 30 min and 1 h did not cause significant deformation, probably because the 3D printing was performed with 100% filling of the prosthesis, producing a solid sphere (Figure 1B).

For all cases described, the surgical technique adopted was transconjunctival enucleation. After preparation of the ocular area and administration of preanesthetic medications (acepromazine, methadone, ceftriaxone, and dexamethasone), the procedure was performed under general anesthesia with propofol and maintenance with isoflurane. The incision was made around the eyeball, followed by dissection of the extraocular muscles and ligation of the optic nerve and blood vessels. Removal of the eyeball was followed by inspection of the orbital cavity and implantation of the 3D prosthesis in the ocular cavity and suturing with remaining conjunctival tissues and extraocular muscles. The surgery was completed with skin suture, reversal of the anesthetic plane, and rigorous postoperative monitoring for detection of complications (2).

## Case reports

All patients in this report presented advanced glaucoma with clinical signs of advanced chronic glaucoma, including buphthalmos and persistent intraocular hypertension, and underwent enucleation with 3D prosthesis implantation. The follow-up descriptions of the cases are described in Table 4.

**Table 4:** Description of the history, diagnosis, treatment, and post-surgical follow-up of dogs with advanced glaucoma undergoing enucleation with 3D prosthesis implantation.

**Case number**	**Breed/age**	**Clinical history**	**Diagnosis**	**Surgical procedure Prosthesis diameter (mm)**	**Post-surgical follow-up**
1	French Bulldog/4.5 years	Perforated corneal ulcer in the RE. Ocular trauma due to failure of protective containment.	Advanced refractory glaucoma and iris exposure.	RE enucleation with implantation of intraorbital 3D prosthesis (20mm)	Complete healing in 20 days. Follow-up after 8 months. No signs of rejection, inflammation, or infection.
2	Pinscher/13.9 years	Bilateral cataract and recurrent episodes of corneal ulcers. Elevated IOP (33 mmHg).	Advanced glaucoma, buphthalmos and irreversible blindness.	LE enucleation with implantation of intraorbital 3D prosthesis (19mm)	Good recovery in 21 days. Follow-up after 6 months. No signs of rejection, inflammation, or infection.
3	French Bulldog/10.2 years	History of hemoparasitic infection and high IOP (41 mmHg in the RE). Undergoing treatment for ehrlichiosis.	Refractory glaucoma, persistent pain, and blindness.	RE enucleation with implantation of intraorbital 3D prosthesis (20mm)	Complete recovery after 25 days with no signs of prosthesis rejection. Positive PCR for *Babesia* spp. followed by specific treatment.
4	German Shepherd/11.1 years	Sudden increase in ocular volume, suspected insect bite. IOP (36 mmHg). Recent diagnosis of ehrlichiosis.	Buphthalmos and irreversible blindness.	LE enucleation with implantation of intraorbital 3D prosthesis (22mm)	*Extrusion of the prosthesis by the animal without supervision, no infectious or inflammatory complications observed. Owners chose not to replace the prosthesis*.
5	Shih- Tzu/11.5 years	Severe glaucoma, apathy, and aggressiveness to manipulation. Undergoing treatment for ehrlichiosis.	Advanced and refractory glaucoma.	LE enucleation with implantation of intraorbital 3D prosthesis (20mm)	Complete healing in 21 days. PCR for *Ehrlichia* spp. negative. Follow-up after 7 months. No signs of rejection, inflammation, or infection.
6	Pinscher/9.8 years	History of a fall down the stairs 8 months ago. Progressive corneal opacification.	Glaucoma secondary to traumatic injury.	LE enucleation with implantation of intraorbital 3D prosthesis (19mm)	Complete healing after 20 days. No need for continued medication and no signs of prosthesis rejection in the evaluation after 6 months.
7	Poodle/5.3 years	Corneal perforation associated with iris hernia. Intense pain.	Advanced glaucoma with iris hernia.	RE enucleation with implantation of intraorbital 3D prosthesis (20mm)	Good recovery in 21 days. Negative PCR for Anaplasma. Follow-up after 5 months. No signs of rejection, inflammation, or infection.
8	Shih-Tzu/16.2 years	History of ocular trauma in the RE, evolution for more than 2 years.	Advanced refractory glaucoma and buphthalmos.	RE enucleation with implantation of intraorbital 3D prosthesis (20mm)	Adequate surgical recovery, without complications. Follow-up after 4 months. No signs of rejection, inflammation, or infection.
9	Shih-Tzu/8.5 years	Trauma due to cat scratch, progressive corneal opacification.	Traumatic glaucoma and irreversible blindness.	LE enucleation with implantation of intraorbital 3D prosthesis (20mm)	Good recovery in 20 days. Follow-up after 4 months. No signs of rejection, inflammation, or infection.
10	Mixed breed/5.4 years	Bilateral glaucoma, ocular hyperemia, hyporexia and apathy.	Advanced glaucoma with lens dislocation.	Bilateral enucleation with implantation of intraorbital 3D prosthesis (Right: 23mm) (Left: 23mm)	Complete healing in 21 days. PCR for *Ehrlichia* spp. negative. Follow-up after 3 months. No signs of rejection, inflammation, or infection.
11	Mixed breed/5.4 years	Corneal perforation with active bleeding. Undergoing treatment for ehrlichiosis and anaplasmosis.	Severe glaucoma and secondary infection.	RE enucleation with implantation of intraorbital 3D prosthesis (20mm)	Complete healing after 20 days. Negative post-treatment PCR. No signs of prosthesis rejection in the evaluation after 2 months.

Pupillary evaluation revealed that six dogs had absent pupillary reflex in at least one eye, indicating damage to the optic nerve secondary to prolonged ocular hypertension. In addition, the Schirmer Tear Test (STT) showed reduced tear production in several cases, probably associated with glaucoma-induced autonomic dysfunction (1). Mature cataract was observed in 27% of patients, reinforcing the relationship between lens opacification and obstruction of aqueous humor outflow, favoring progression to intraocular hypertension and secondary glaucoma (7). Lens dislocation was also a relevant finding, confirming its impact as a predisposing factor for increased IOP and worsening of the clinical condition (20).

Hemoparasitic infections was diagnosed in 36,6% of dogs (cases 3/4/5 and 11), with ehrlichiosis and babesiosis as the predominant infections. The presence of these diseases may be associated with ocular inflammatory processes that contribute to the refractoriness of glaucoma to clinical treatment (3, 21). In these cases, the administration of doxycycline helped in the recovery and control of ocular inflammation, demonstrating a beneficial effect on the postoperative evolution (3).

As observed in Table 4, the causes of advanced glaucoma varied among the animals in this case series. The pathological condition may develop due to various injuries to the eyeball, such as cat scratches, insect bites, falls, and traumatic punctures. In addition, glaucoma can also be corrected with infectious diseases and other ocular pathologies, such as corneal ulcers and hemoparasitic infections. No direct correlation with breed, weight, or age was observed. The average age of the dogs was 9.75 years, ranging from 4.5 to 16.2 years. The average weight was 12.27 kg, with a minimum of 2.0 kg and a maximum of 40.2 kg (Tables 1, 4).

**Figure 2:**
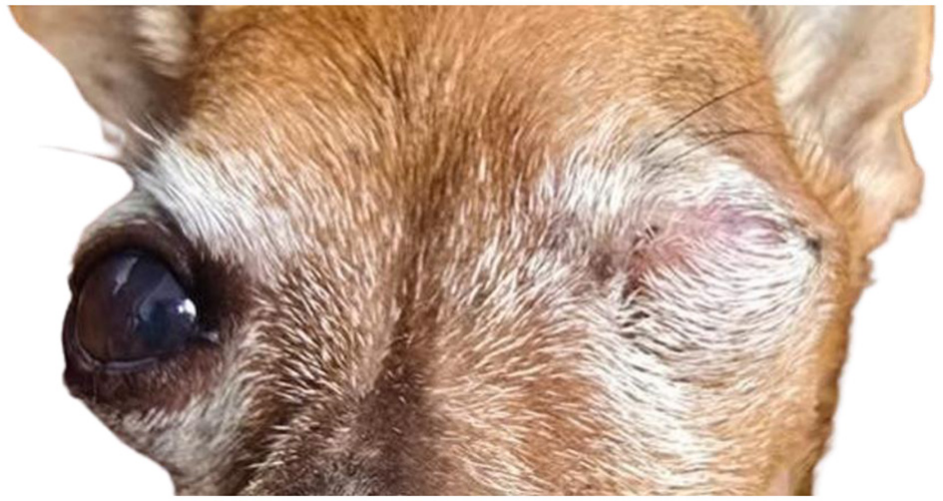
Photographic record of case 2, a 13.9-year-old Pinscher dog with bilateral cataract, advanced glaucoma, and irreversible blindness. Enucleation of the left eye was performed with implantation of the 3D prosthesis, record made after 6 months of implantation without signs of inflammation, infection, or secretions. Complete healing with removal of the suture was performed after 21 days, the animal presented calm behavior without signs of pain or pruritus.

For this case series, 12 eyeball prosthesis implants were analyzed. The adaptation to the 3D prostheses was satisfactory, with 91% of the 11 dogs (case 10—bilateral implant), showing complete healing after around 21 days, when the sutures were removed, and no signs of acute inflammation or rejection of the implant were observed (Figure 2). Only one patient had prosthesis extrusion due to early removal of the Elizabethan collar without supervision, highlighting the importance of environmental control and adherence to the postoperative protocol (8). Even with forced removal of the implant, there was no subsequent infection or inflammation.

**Figure 3:**
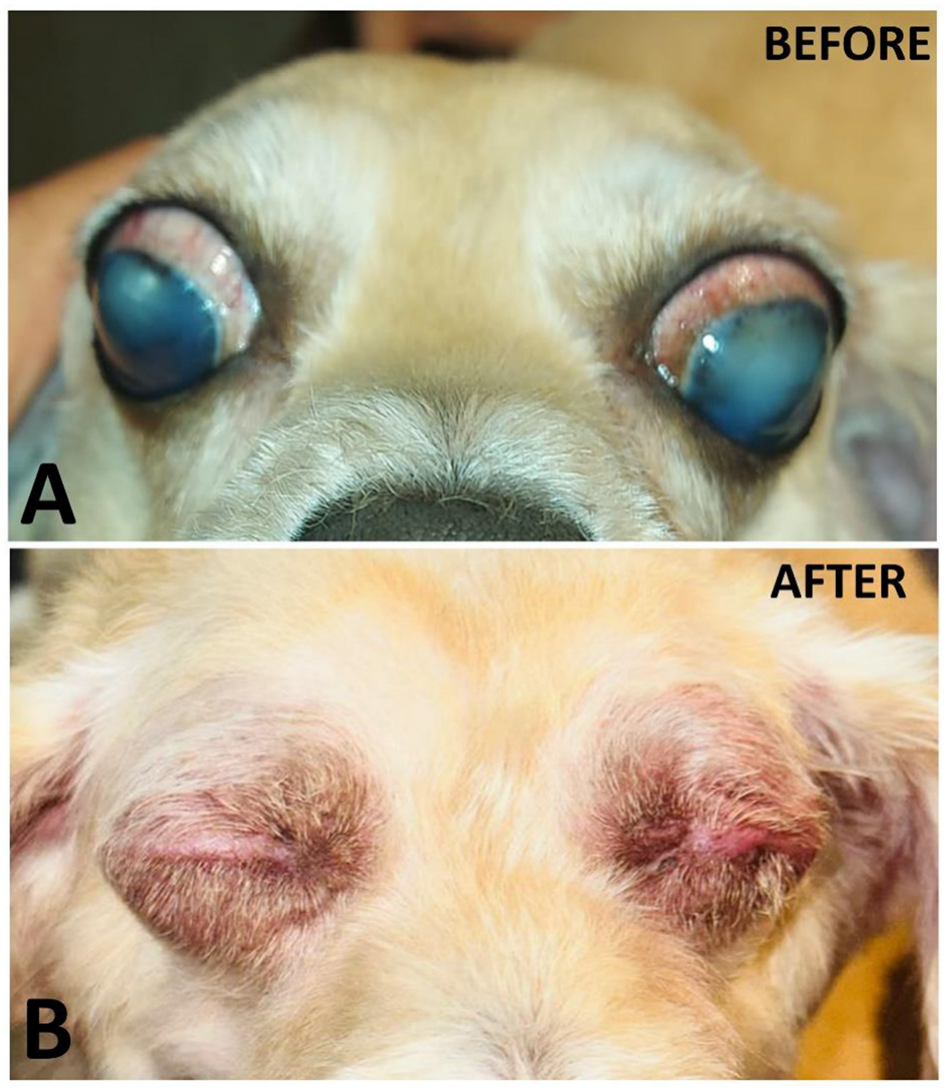
Photographic records of case 10, a 5.4-year-old mixed breed dog with bilateral advanced glaucoma with lens dislocation and severe pain. **(A)** Photographic record before the surgical procedure. **(B)** 3 months after bilateral enucleation with implantation of the 3D prosthesis. No signs of rejection, inflammation, or infection were observed. The animal presented calm behavior, with no signs of pain or pruritus.

The patients in this report are still being evaluated on an ongoing basis. The first prostheses were implanted in mid-2024, and at the time of writing this report (8 months after the first implant), no signs of rejection, pain, inflammation, or infection associated with the implant were observed. The animals presented adequate healing, with satisfactory hair coverage over the suture area (Figure 3). With the 3D prosthesis, the ocular cavity was occupied and there was a lower chance of accumulation of secretions, changes in the eyelids and tearing, and anatomical deformities in the long term. It is also worth noting that the 3D prosthesis provides a better aesthetic result for the animals compared to enucleation without implant, which reflects in greater emotional comfort for the guardians, who accepted the enucleation more easily.

## Discussion

The analysis of the eleven clinical cases allowed the identification of recurrent patterns and challenges in the surgical management of dogs with advanced glaucoma, often associated with hemoparasitic infections and other severe ophthalmic disorders. The decision for enucleation was based on the refractoriness to medical treatment, the presence of intense ocular pain, and the progressive deterioration of visual function. Recent clinical studies reinforce that advanced-stage canine glaucoma is commonly associated with severe intraocular hypertension, buphthalmos, and irreversible vision loss, making enucleation a necessary therapeutic alternative in many cases (22, 23).

The evaluated canine patients exhibited clinical signs consistent with refractory glaucoma, including marked buphthalmos, episcleral congestion, and absent or diminished pupillary reflexes. In some cases, corneal perforation, iris prolapse, and a history of ocular trauma were observed, representing predisposing factors for disease progression. The concomitant presence of hemoparasitic infections, such as ehrlichiosis, anaplasmosis, and babesiosis, was a relevant finding, highlighting the correlation between systemic infections and ocular inflammation that can exacerbate intraocular hypertension and impair the therapeutic response (21, 38).

Mature bilateral cataract was another common finding, reinforcing the association between lens opacification and predisposition to increased intraocular pressure. In such cases, the progression to glaucoma may be related to obstruction of aqueous humor outflow resulting from lens luxation, a condition observed in one of the individuals in this study. Experimental and retrospective studies have confirmed that lens luxation can act as a mechanical barrier to aqueous humor drainage, thereby worsening intraocular hypertension and necessitating surgical intervention (38).

In the cases described, previous treatments included hypotensive eye drops containing dorzolamide, brinzolamide, and timolol, which showed initial efficacy but progressive loss of effect as the disease advanced. This phenomenon aligns with clinical reports describing the limited effectiveness of topical agents in controlling advanced glaucoma due to irreversible optic nerve damage and structural alterations of the anterior chamber (38, 39).

The enucleation technique combined with the implantation of 3D printed orbital prostheses proved to be feasible and safe in most of the analyzed cases. Enucleation provided definitive resolution of pain associated with refractory glaucoma, while the orbital implant played a primarily reconstructive role, contributing indirectly to postoperative comfort, prevention of conjunctival contraction, and improved cosmetic and behavioral outcomes (22–25).

Polylactic acid (PLA) proved to be a suitable material due to its biocompatibility, dimensional stability, and mechanical strength (26, 27). The customization enabled by 3D printing technology allowed for better anatomical adaptation of the prosthesis, promoting tissue integration, and reducing postoperative complications, in addition to providing satisfactory aesthetic results (12, 25).

Historically, various materials have been employed for orbital reconstruction in dogs and cats, including glass, silicone, porous polyethylene, and hydroxyapatite. Glass and silicone implants, although still used in limited contexts, show poor tissue integration and a higher risk of extrusion and infection (24, 28). In contrast, hydroxyapatite has become one of the most biocompatible options, promoting fibrovascular ingrowth and long-term stability; however, its high cost and the risk of exposure in cases of infection limit its routine application (22, 29).

Comparatively, biodegradable polymers such as polylactic acid (PLA) and polycaprolactone (PCL) have gained prominence due to their controllable physicochemical properties and lower production costs (12, 16). Recent studies in both human and veterinary medicine have shown that PCL, although more resistant to thermal deformation, exhibits a slower degradation rate than PLA, which may delay tissue integration (30). Conversely, PLA, when properly processed and sterilized, offers a favorable balance between mechanical stability and biodegradability, making it widely used in 3D-printed medical devices (26, 27).

Beyond the material itself, the shape and porosity of the implants play a decisive role in the biological response. Porous implants, such as those made of hydroxyapatite and expanded polyethylene, promote fibrovascular ingrowth and tissue anchorage, thereby reducing the risk of migration, although they present greater susceptibility to bacterial colonization if aseptic conditions are compromised (24, 25). In contrast, solid PLA or PCL implants provide lower tissue integration but facilitate cleaning and infection control, making them suitable for immunosuppressed patients or those with a history of orbital infection (12, 22).

Regarding the manufacturing method, conventional implants, manually molded, are associated with higher costs and poorer anatomical conformity. In contrast, 3D printing enables the fabrication of patient-specific prostheses based on imaging data, ensuring greater volumetric precision, and improved cosmetic outcomes. Experimental studies have demonstrated that the use of additive manufacturing for orbital implant fabrication significantly reduces costs and surgical time, while enhancing anatomical adaptation and owner satisfaction (12, 22).

Recent economic and clinical evaluations also indicate that fabrication of 3D prostheses can reduce operational costs by up to 70% compared to commercial models, while maintaining favorable aesthetic and functional outcomes (25, 31). This cost reduction, combined with the availability of affordable printers and biocompatible materials such as PLA, makes this technology particularly attractive for veterinary hospitals with limited infrastructure (26, 27).

When compared with conventional methods of orbital reconstruction, PLA prostheses offer relevant logistical advantages: they can be sterilized by autoclaving, stored for long periods, and rapidly customized to individual anatomical needs. Recent studies confirm the dimensional stability of PLA after autoclave sterilization and the maintenance of its mechanical and biocompatibility properties, establishing it as a safe option for veterinary use (32). This versatility is particularly important in clinical contexts with limited resources, where access to medical-grade materials and industrial printers remains restricted (26, 33).

From a biological standpoint, the complication rates observed in this study were comparable or lower than those reported for hydroxyapatite and PCL implants in dogs and humans (12, 30). No cases exhibited significant inflammatory reaction, infection, or rejection, reinforcing the safety of PLA when properly sterilized and implanted under aseptic technique. Similar outcomes were reported by Palmer et al. (22), who observed high owner satisfaction and a low incidence of post-enucleation complications, and by Diaz Bujan et al. (23), who documented favorable behavioral adaptation following the procedure.

Recent literature also highlights the potential of additive manufacturing to replace traditional prototyping techniques, allowing for intraoperative adjustments and preoperative anatomical simulations. This flexibility is particularly useful in complex cases, such as orbits deformed by trauma or neoplasia (12, 25). Furthermore, experimental studies indicate that combining polymers with bioceramics—such as hydroxyapatite or β-tricalcium phosphate—results in hybrid implants with optimized mechanical and biological properties, enhancing structural strength and tissue integration (16, 29).

Despite the advances achieved, some challenges remain. The hydrolytic degradation of PLA can generate acidic by-products that, over time, may alter the local pH and delay tissue healing (16, 26). Long-term follow-up studies, such as those by Park et al. (12) and Valencia (34), are essential to monitor structural changes, stability, and the potential need for prosthesis replacement. Another relevant aspect to consider is imaging interference, as both PLA and PCL are radiotransparent, which may hinder monitoring through conventional radiography, although they do not interfere with ultrasonography or magnetic resonance imaging (25, 35).

Therefore, the comparison among materials demonstrates that PLA represents a favorable balance between cost, ease of fabrication, biocompatibility, and clinical applicability. Three-dimensional printing emerges not only as a viable alternative but also as a significant advancement over conventional methods, integrating precision, safety, and accessibility. The adoption of this technology, combined with professional training and strict sterilization protocols, has the potential to transform the management of orbital cavities in veterinary ophthalmology, reducing costs and improving patient quality of life (22, 25, 26).

## Conclusions

This case series highlights the feasibility and clinical potential of three-dimensional (3D) printing technology for developing customized orbital implants in dogs with advanced glaucoma undergoing enucleation. The use of biocompatible polylactic acid (PLA) implants provided good postoperative adaptation, comfort, and favorable cosmetic outcomes, with no inflammatory or infectious complications observed during an eight-month follow-up. Compared with other biodegradable polymers, PLA offers advantages such as controlled degradation rate, dimensional stability, low cost, and compatibility with standard sterilization methods. Despite these promising results, further studies with larger samples and longer follow-up are required to evaluate the long-term degradation profile, biomechanical stability, and overall safety of PLA orbital implants in veterinary ophthalmology.

## Data Availability

The original contributions presented in the study are included in the article/supplementary material, further inquiries can be directed to the corresponding author.
